# 2-Keto-d-Gluconate-Yielding Membrane-Bound d-Glucose Dehydrogenase from *Arthrobacter globiformis* C224: Purification and Characterization

**DOI:** 10.3390/molecules20010846

**Published:** 2015-01-08

**Authors:** Qing Xue, Zhuan Wei, Wenjing Sun, Fengjie Cui, Silian Yu, Qiang Zhou, Jingze Liu

**Affiliations:** 1 School of Food and Biological Engineering, Jiangsu University, Zhenjiang 212013, China; E-Mail: xueqing1114@126.com; 2 College of Life Science, Hebei Normal University, Shijiazhuang 050016, China; E-Mail: liujingze@mail.hebtu.edu.cn; 3 Parchn Sodium Isovitamin C Co. Ltd, Dexing 334221, China; E-Mails: weizhuan8856@163.com (Z.W.); ysl@parchn.com (S.Y.); zhouqiang@parchn.com (Q.Z.); 4 Jiangxi Provincial Engineering and Technology Center for Food Additives Bio-production, Dexing 334221, China; 5 Department of Pharmaceutical, Hebei Chemical and Pharmaceutical College, Shijiazhuang 050026, China

**Keywords:** *Arthrobacter globiformis*, 2-ketogluconic acid (2KGlcA), d-glucose dehydrogenase (GlcDH), purification, enzymatic properties

## Abstract

Glucose dehydrogenase (GlcDH) is the rate-limiting catalyst for microbial conversion of glucose to the important organic acid 2-ketogluconic acid (2KGlcA). In this study, a d-glucose dehydrogenase was purified from the industrial 2KGlcA producer *Arthrobacter globiformis* C224. After four purification steps, the GlcDH was successfully purified over 180 folds and specific activity of 88.1 U/mg. A single protein band of 87 kDa was detected by SDS-PAGE. The purified GlcDH had the broad substrate specificity with the *K*_m_ values for d-glucose, d-xylose, d-galactose and maltose of 0.21 mM, 0.34 mM, 0.46 mM and 0.59 mM, respectively. The kinetic studies proved that *A. globiformis* GlcDH followed the ping-pong kinetic mechanism. The GlcDH showed an optimum catalytic activity at pH 5.0 and 45 °C with the stable activity at temperature of 20–40 °C and pH of 6.0–7.0. Organic solvents, metal ions or EDTA could significantly influence the GlcDH activity to different degrees.

## 1. Introduction

Erythorbic acid is an effective food antioxidant approved as a generally recognized as safe (GRAS) substance [1]. Currently, annual worldwide erythorbic acid production is estimated at 40,000 tons and over 80% is produced in China [2]. Industrial erythorbic acid production includes a two-step process including microbial conversion of d-glucose to 2-ketogluconic acid (2KGlcA) and chemical lactonization of 2KGlcA to the target product erythorbic acid [1,2]. Microorganisms from genera *Pseudomonas*, *Serratia*, *Gluconobacter* and *Acetobacter* have been reported as the potential origins of 2KGlcA producers [3,4,5,6]. Some stains including *P. fluorescens* AR4 and *A. globiformis* C224 with the high glucose tolerance of over 140 g/L and a 2KGlcA yield of over 92.80% have been screened by our group and are currently applied by most Chinese erythorbic acid companies [7,8].

The microbial 2KGlcA production includes two consecutive periplasmic oxidation reactions mediated by glucose dehydrogenase (GlcDH, EC 1.1.5.2) and gluconate dehydrogenase (GADH) to convert the substrate glucose into the target product 2KGlcA [9]. In most cases, the conversion of glucose to gluconic acid by GlcDH is the rate-limiting step. GlcDH catalyzes d-glucose into gluconate by transfer of electrons to ubiquinol oxidase through ubiquinone. It has been found that the steady-state-type kinetics of membrane-integrated quinoprotein glucose GlcDH followed the ping-pong mechanism. Mg^2+^ anchors PQQ to the membrane-bound GlcDH protein and activates the bound cofactor, which benefits the enzyme toward the sugars tested [10].

There are various published references about purification and catalytic characteristics of GlcDHs from the bacteria *E. coli* [11], *G. suboxydans* [12], *B. thuringiensis* [13], *Pseudomonas* sp. [14] and *A. calcoaceticus* [15]. The majority of these GlcDHs is known as monomers with mass ranging from 87 kDa to 90 kDa, and possesses the broad substrate specificity for various monosaccharides and an optimum catalytic pH value of 6.0 [11,12,14]. GlcDHs from *B. thuringiensis* and *A. calcoaceticus* have been proven to be peculiar, being formed by two subunits and an optimum catalytic activity at pH 8.0 [13,15]. However, very few studies about the GlcDHs purified from 2KGlcA producers, or involved in 2KGlcA production are known.

The *A. globiformis* C224 strain is the industrial 2KGlcA producer now applied for erythorbic acid production by Parchn Sodium Isovitamin C Co., Ltd. *A. globiformis* C224 has the significant 2KGlcA production performance with a stable 2KGlcA concentration of 124.74 g/L, average volumetric productivity of 11.23 g/L·h and yield of 0.97 g/g in a continuous process [8]. However, the references related to the purification and characteristic analysis of the membrane-bound d-glucose dehydrogenase (GlcDH) from *A. globiformis* C224 are still not available. To further improve 2KGlcA production performance and develop the *A. globiformis* strains, the present study will aim to: (1) first purify the GlcDH from the membrane fraction of 2KGlcA industrial strain *A. globiformis* C224; and (2) characterize the biochemical and catalytic properties of the purified GlcDH.

## 2. Results and Discussion

### 2.1. Extraction and Purification of GlcDH from A. globiformis C224

Membrane proteins are embedded in the mosaic lipid bilayer with low contents and hydrophobic characteristics, which will hinder the extraction of the interested membrane proteins [16]. Some kinds of detergents including sodium dodecyl sulfate (SDS), bile acid salts, nonionic detergents and zwitterionic detergents can soluble the membrane proteins without affecting their structural features [17,18]. Triton X-114 is a nonionic detergent used for extracting the membrane-associated proteins [19,20,21] including the cell envelope associated proteins from *M. tuberculosis* [22] or membrane-associated proteins from *M. bovis* [23]. In this study, approximate 9758.1 mg of crude membrane-protein with the GlcDH activity 4730.6 U was extracted from 8.910 g of *A. globiformis* C224 cells at 0 °C with the ultrasonic and 1% Triton X-114 assistance (Table 1). With the increase of storage temperature to 37 °C, the extracted solution was partitioned into Triton X-114 and aqueous phases. After centrifugation, 6004.5 mg of membrane-protein was recovered from Triton X-114 phase with the increased GlcDH specific activity of 0.7 U/mg and 1.5-fold purification.

**Table 1:** Summarization of the GlcDH purification from *A. globiformis* C224.

Step	Protein (mg)	Enzyme Activity (Units)	Specific Activity (Units/mg)	Recovery (%)	Purification (Fold)
Membrane fraction	9758.1 ± 121.3	4730.6 ± 96.5	0.5	100.0	1.0
Triton X-114 phase separation	6004.5 ± 96.1	4396.4 ± 79.8	0.7	92.9	1.5
Acetone precipitation	1103.7 ± 30.6	1524.9 ± 34.2	1.4	32.2	2.9
PEG precipitation	408.8 ± 17.4	846.3 ± 12.3	2.1	17.9	4.3
Ethanol precipitation	33.6 ± 1.2	268.2 ± 6.4	7.9	5. 7	16.6
Hydroxylapatite fraction	1.8 ± 0.1	159.4 ± 2.1	88.1	3.4	183.5

**Figure 1:**
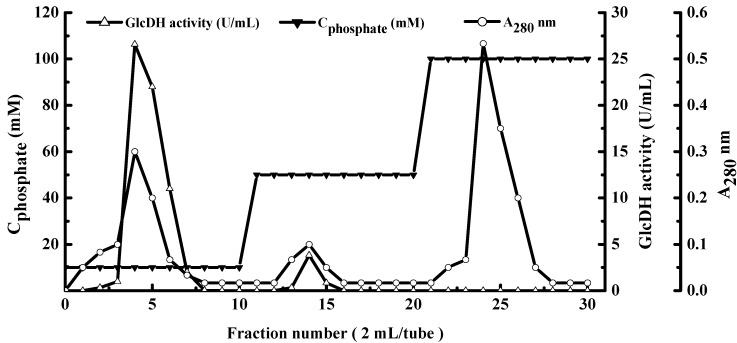
Elution profiles of GlcDH from *A. globiformis* C224 by hydroxylapatite column chromatography. Mobile phase: equilibrium liquid: buffer D; elution: buffer D, buffer E, buffer F.

As shown in Table 1, the consecutive precipitation steps with acetone, polyethylene glycol 6000 and ethanol yielded 33.6 mg of purified GlcDH with the increased specific activity of 7.9 U/mg and 16.6-fold purification. Figure 1 presents the hydroxyapatite column chromatography profile. The fraction eluted with buffer D had the highest GlcDH activity of 10.62 U/mL. The column purification yielded 1.8 mg of purified GlcDH with a maximum specific activity of 159.4 U/mg and 183.5-fold purification, which were higher than GlcDH from *G. suboxydans* with a specific activity 35.0 U/mg and 23.3-fold purification [12].

### 2.2. Type Identification of GlcDH from A. globiformis C224

**Figure 2:**
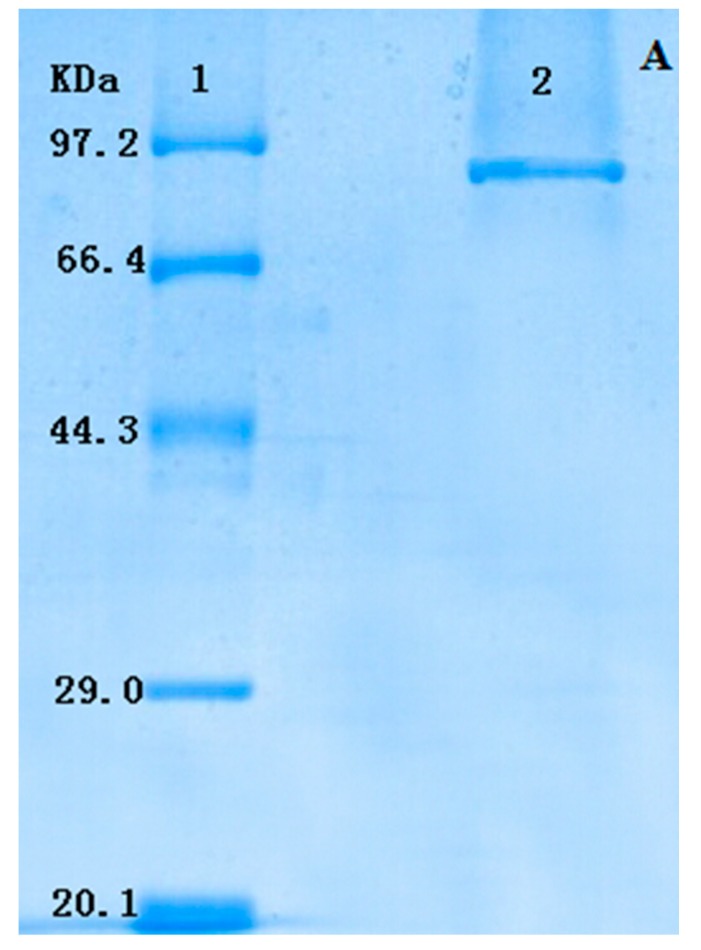

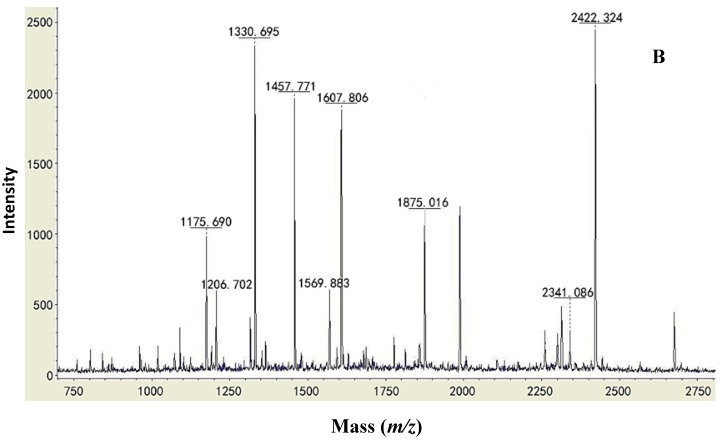
Type identification of GlcDH from *A. globiformis* C224. (**A**) SDS-PAGE of GlcDH from *A. globiformis* C224. Lane 1: standard marker proteins: phosphatase b (97,200 Da), bovine serum albumin (66,409 Da), ovalbumin (44,287 Da), carbonic anhydrase (29,000 Da) and trypsin inhibitor (20,100 Da). Lane 2: purified GlcDH; (**B**) Mass spectra of peptides of GlcDH from *A. globiformis* C224 by MALDI-TOF-MS.

The purified GlcDH showed a single band on SDS-PAGE which indicated that it had the high purity without subunits (Figure 2A). The molecular weight of GlcDH in SDS-PAGE was estimated to be 87 kDa. The GlcDHs purified from *E. coli* [11] and *Pseudomonas* sp. [14] on the SDS-PAGE had one band, while others from *B. thuringiensis* [13] and *A. calcoaceticus* [15] had two subunits.

Partial sequences of purified GlcDH from *A. globiformis* C224 were further analyzed by applying the protein band in the SDS-PAGE for MALDI-TOF-MS and NCBI BLAST search. The mass spectrum of GlcDH peptides is shown in Figure 2B. 

The similar sequences in the database of the NCBI blast, predicted molecular mass and score of the GlcDH are shown in Table 2. The molecular mass of GlcDH was determined as approximately 87 kDa, which agreed with that obtained from SDS-PAGE. From Table 2, the GlcDH from *A. globiformis* C224 had the similar amino acid sequence to GlcDHs from *Ps. entomophila* L48 and *Ps. putida* GB-1 with the high scores of 142 and 114, respectively.

**Table 2:** Database search results of GlcDH from *A. globiformis* C224.

Protein	Similar Sequence in the Database of the NCBI Blast	Strains	Mass (Da)	Score
Gi**|**104780479 glucose dehydrogenase	R.LLALDPDTGAEIWR.Y	*Pseudomonas entomophila* L48	87,067	142
	R.GIGPFTAGGYYSTSPAAITR.S			
Gi**|**339489095 glucose dehydrogenase	R.TEHGDRYSPLR.Q	*Pseudomonas putida* S16	86,935	117
	R.LLALDPDTGAEIWR.F			
	R.GVSYYDENRYVSR.D			
Gi**|**167035354 PQQ-dependent glucose dehydrogenase	R.TEHGDRYSPLR.Q	*Pseudomonas putida* GB-1	86,939	114
	R.LLALDPDTGAEIWR.F			
	R.GVSYYDENRYVSR.D			
Gi**|**26988177 glucose dehydrogenase	R.LLALDPDTGAEIWR.Y	*Pseudomonas putida* KT2440	86,926	100
	R.GVSYYDENRYVSR.D			

### 2.3. Enzymatic Properties of Purified Membrane-Bound GlcDH from A. globiformis C224

#### 2.3.1. Substrate Specificity

The substrate specificity of the crude membrane protein and purified GlcDH from *A. globiformis* C224 was summarized in Table 3. Purified GlcDH showed a broad substrate specificity with different monosaccharide including d-glucose, maltose, d-galactose, d-xylose or d-arabinose as the substrates. However, compared with the catalytic capacity using glucose as the substrate, other substrates found to be catalytic active with the *A. globiformis* GlcDH were maltose (17%), d-galactose (20%), d-xylose (22%) and d-arabinose (8.0%), thus indicating that d-glucose is the preferred substrate of *A. globiformis* GlcDH. Similar results also were found that the GlcDH from *G. suboxydans* could catalyze d-glucose or maltose [12].

**Table 3:** Substrate specificity of GlcDH from *A. globiformis* C224.

Substrate	Concentration of Substrate (mM)	Membrane Enzyme (U/mL)	Purified EnzymeRelative Activity (%)
d-Glucose	33.0	21.9 ± 0.9	100.0 ± 1.6
d-Gluconate	33.0	82.2 ± 3.8	0
Maltose	33.0	3.1 ± 0.1	17.0 ± 0.8
d-Sorbose	33.0	0	0
d-Galactose	33.0	3.8 ± 0.1	20.0 ± 0.9
d-Mannose	33.0	0	0
d-Fructose	33.0	0	0
d-Arabinose	33.0	2.1 ± 0.1	8.0 ± 0.5
Malic acid	33.0	0	0
Sucrose	33.0	0	0
Citric acid	33.0	0	0
d-Xylose	33.0	5.3 ± 0.2	22.0 ± 0.9

#### 2.3.2. Kinetic Studies of GlcDH from *A. globiformis* C224

##### Single Substrate Kinetics

**Figure 3:**
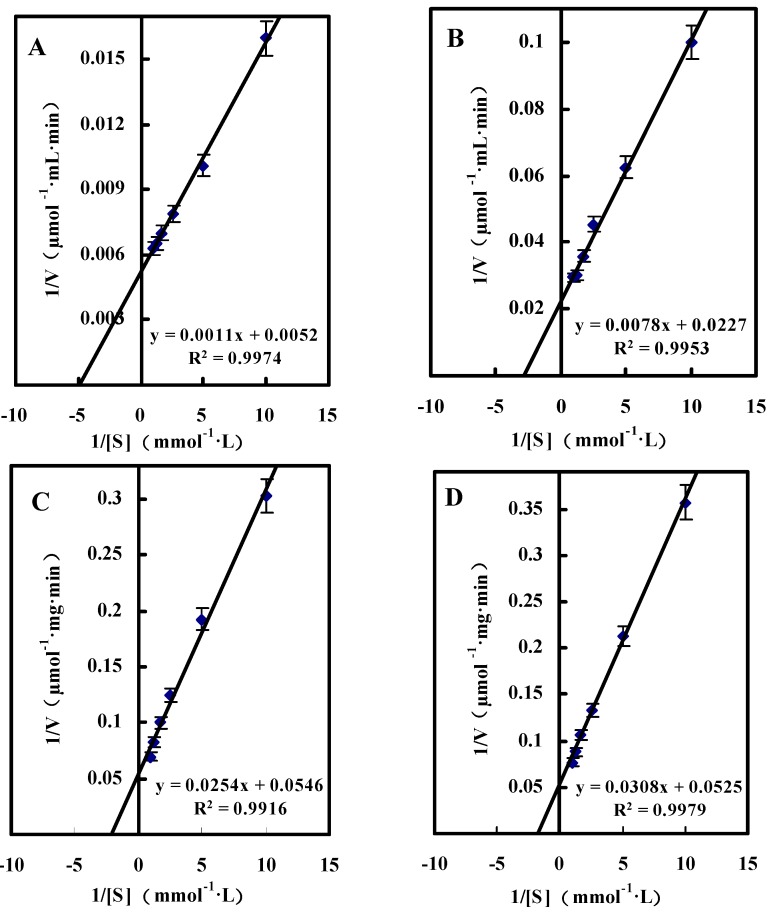
Lineweaver-Burk double reciprocal plots for GlcDH purified from *A. globiformis* C224. (**A**): d-Glucose as substrate; (**B**): d-Xylose as substrate; (**C**): Maltose as substrate; (**D**) d-Galactose as substrate.

Figure 3 shows the Lineweaver-Burk double reciprocal plotting profiles of d-glucose, d-xylose, maltose or d-galactose as substrates. Based on the Lineweaver-Burk double reciprocal plots, the *K*_m_ and V_max_ values for d-glucose, d-xylose, d-galactose and maltose were showed in Table 4, which verified the conclusion that the optimum substrate for *A. globiformis* GlcDH was d-glucose. Some GlcDHs purified from *A. calcoaceticus* and *B. cereus* had the *K*_m_ values of 22 mM and 7 mM for glucose [15,24], which were significantly higher than those obtained in the present study.

**Table 4:** Kinetic parameters of GlcDH for various substrates.

Substrate	*K*_m_ (mM)	V_max_ (μmol/mg·min)
d-Glucose	0.21 ± 0.01	192.31 ± 9.45
d-Xylose	0.34 ± 0.02	44.05 ± 1.94
d-Galactose	0.46 ± 0.02	18.32 ± 0.76
Maltose	0.59 ± 0.03	19.05 ± 0.85

##### Dual Substrates Kinetics

**Figure 4:**
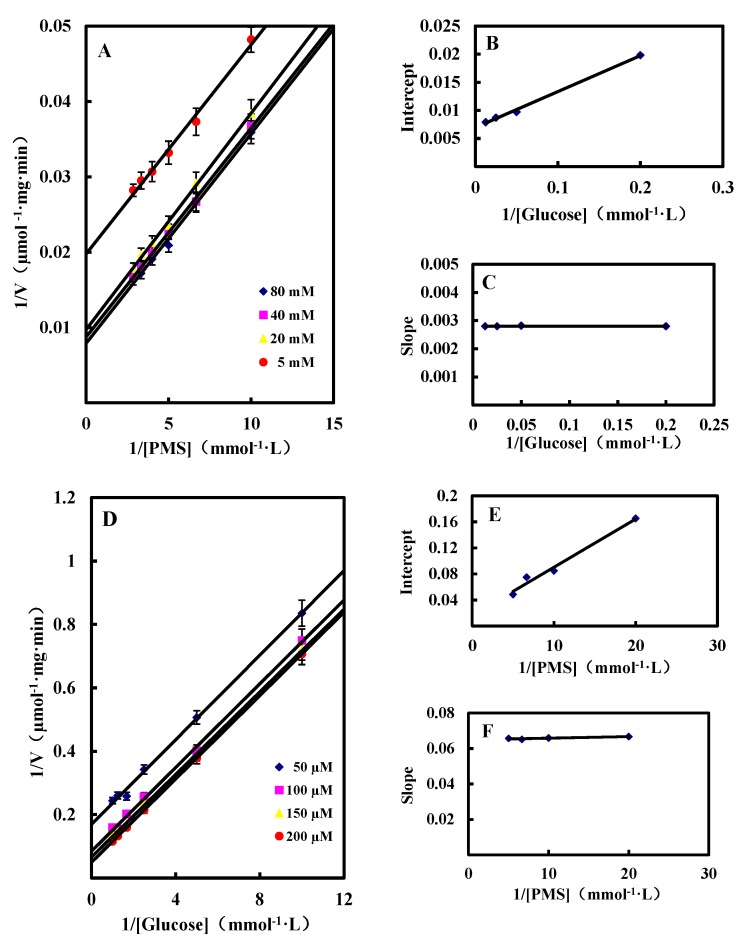
Initial velocity patterns for GlcDH reaction from *A. globiformis* C224. (**A**) PMS was varied at the following fixed concentrations of d-glucose: 5, 20, 40 and 80 mM; (**D**) d-Glucose was varied at the following fixed concentrations PMS: 50, 100, 150 and 200 µM. Inset (**B**), (**C**), (**E**) and (**F**), slope and 1/v axis intercept *versus* 1/[Glucose] or 1/[PMS].

In the present study, *N*-methylphenazonium methyl sulfate (PMS) was used as the artificial electron acceptor to investigate the dual substrates kinetics mechanism of GlcDH. By changing the concentrations of glucose and PMS, the kinetic profiles by plotting the reciprocal of the initial velocity (1/v) against the reciprocal of one substrate (1/[S]) were obtained as shown as the straight lines in Figure 4. GlcDH showed ping-pong behavior due to the parallel plotted lines of initial reaction rates. Similar ping-pong kinetic mechanisms were also observed in the immobilized lipase [25] and tRNA-guanine transglycosylase from *E. coli* [26].

By calculation, the *K*_m_ values for glucose and PMS were estimated as 9.2 mM and 0.4 mM and V_max_ was 140.11 μmol/mg·min, respectively. The affinity of GlcDH towards PMS seems to be higher than that towards glucose since *K*_m(P__MS)_ of 0.4 mM was significantly lower than *K*_m(glucose)_ of 9.2 mM. Hence, the catalytic reaction began from the bind of PMS to *A. globiformis* GlcDH, and then transferred the O_2_ to the bound glucose to produce gluconic acid.

##### Inhibition by Gluconic Acid

Inhibition types were obtained by calculating the inhibition parameters from competitive or uncompetitive inhibition equations. The calculated inhibition kinetics terms *K*_m_, V_max_ and the inhibition constant were 7.6 mM, 112.75 µmol/mg·min and 14.8 mM for glucose, and 1.4 mM, 136.27 µmol/mg·min and 11.4 mM for PMS, respectively. According to the lower *K*_m(P__MS)_ value, it could be conformed that the higher affinity of the GlcDH towards PMS than that to glucose.

**Figure 5:**
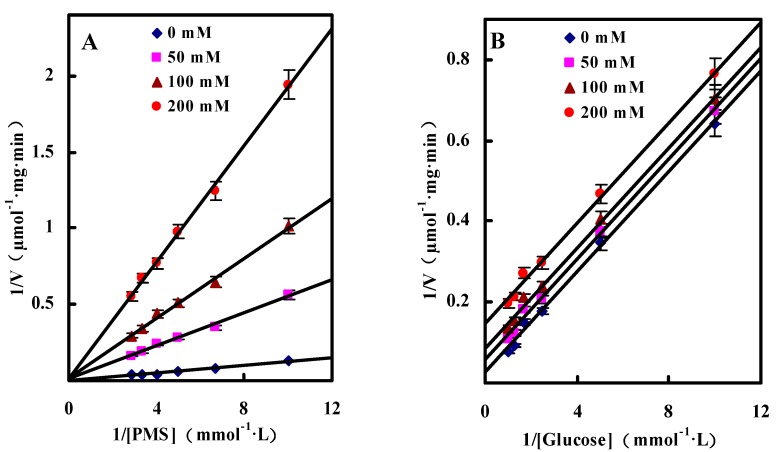
The kinetics of inhibition of the GlcDH reaction by gluconic acid. (**A**) 1/v *versus* 1/[PMS] at 33 mM glucose and the following fixed concentration of gluconic acid: 0 mM, 50 mM, 100 mM and 200 mM; (**B**) 1/v *versus* 1/[glucose] at 100 µM PMS and the following fixed concentration of gluconic acid: 0 mM, 50 mM, 100 mM and 200 mM.

Generally, the catalytic product can affect the activity of enzymes by competitive, noncompetitive or uncompetitive ways. The competitive model had the lines converging at the same point on the y-axis on the Lineweaver-Burk double reciprocal plots, noncompetitive model had the lines converging at the same point but not on the y-axis, while typical uncompetitive model had a series of parallel lines. As shown in Figure 5, the Lineweaver-Burk double reciprocal plots of *A. globiformis* GlcDH were the typical parallel with glucose as the substrate lines and converging lines with PMS as the substrate when gluconic acid was used as the inhibitor at the concentration from 0 to 200 mM. Hence, it could be concluded that the gluconic acid was anon-competitive inhibitor to glucose and a competitive inhibitor to PMS.

#### 2.3.3. Effect of pH on GlcDH Activity and Stability

**Figure 6:**
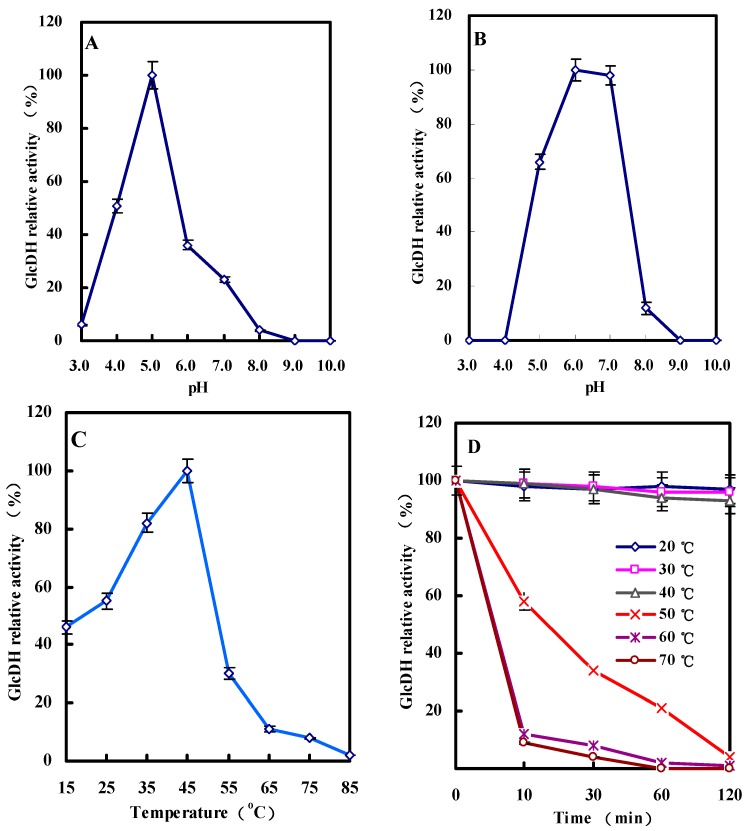
Effects of pH and temperature on the GlcDH activity from *A. globiformis* C224. (**A**) Optimum pH of GlcDH; (**B**) pH stability of GlcDH; (**C**) Optimum temperature of GlcDH; (**D**) Temperature stability of GlcDH.

Figure 6A,B show the influence of pH on the GlcDH activity and stability. From Figure 6A, the highest GlcDH activity appeared at pH 5.0. The GlcDH relative activity decreased to approximately 51% or 36% when the pH dropped to 4.0 or increased to 6.0. Other GlcDHs from *B. thuringiensis* M15 [13], *G. suboxydans* [12], *Pseudomonas* sp*.* [14] and *E. coli* [11] had the different optimal pHs with the values of 8.0, 3.0, 4.5 and 7.0 (Table 5), respectively. Figure 6B also demonstrated that the purified *A. globiformis* C224 GlcDH had the stable activity at pH values between 6.0 and 7.0 after two days storage at 4 °C, and decreased to near zero at pH 4.0 and 9.0.

**Table 5:** Properties of GlcDHs from various microorganisms.

Properties	*Escherichia coli* [11]	*Gluconobacter Suboxydans* [12]	*Bacillus Thuringiensis* [13]	*Pseudomonas* sp. [14]	Acinetobacter Calcoaceticus [15]	Arthrobacter Globiformis(This Study)
Molecular mass of subunits (Da)	88,000	87,000	25,000 26,000	90,000	47,500 48,000	87,000
Optimum pH with ferricyanide	3.5	3.0	ND	4.5	ND	5.0
With DCIP-PMS	6.0	6.0	8.0	6.0	6.0	ND
Optimum temperature (°C)	ND	ND	55	ND	ND	45
Substrate range	Glucose; Mannose; Galactose; Fructose; Rhamnose; Xylose	Glucose; Maltose	Glucose; 2-d-deoxy-d-glucose	Glucose; Mannose; Galactose; Xylose; Maltose; Rhamnose	Glucose; Xylose; Arabinose; Lactose; Galactose; Melibiose; Cellobiose; Maltose	Glucose; Xylose; Galactose; Maltose
*K*_m_ (mM)	ND	ND	14 (Glucose, pH 8.0); 12.2 (2-d-deoxy-d-glucose, pH 8.0)	0.69 (Glucose, pH 4.5); 1.6 (Glucose, pH 6.0)	22 (Glucose, pH 6.0)	0.21 (Glucose, pH 5.0); 0.34 (Xylose, pH 5.0); 0.46 (Galactose, pH 5.0); 0.59 (Maltose, pH 5.0)

ND: not determined.

#### 2.3.4. Effect of Temperature on GlcDH Activity and Stability

Eight reaction temperatures ranging from 15 °C to 85 °C were used for investigating its effect on *A. globiformis* GlcDH activity. In Figure 6C, GlcDH reached to the maximum activity (relative activity of 100%) with the increase of reaction temperature from 15 to 45 °C, and followed the sharp decline of activity to 30% at 55 °C, and even no activity at 85 °C. Hence, a temperature of 45 °C could be selected for maximize the *A. globiformis* C224 GlcDH-catalyzing reaction efficiency. Other GlcDHs such as *B. thuringiensis* GlcDH had the higher reaction temperature of 55 °C to catalyze glucose to produce gluconic acid [13].

The purified *A. globiformis* GlcDH was kept at temperatures from 20 to 70 °C for 10–120 min. As shown in Figure 6D, low temperature of below 40 °C had the stable and highest GlcDH activity after kept for 120 min. Increase of storage temperature to over 50 °C inhibited the GlcDH activity of above 58% after even 10 min treatment. No GlcDH activity was detected after incubation of GlcDH for 60 min at 60 or 70 °C.

#### 2.3.5. Effect of Organic Solvents on GlcDH Activity

**Figure 7:**
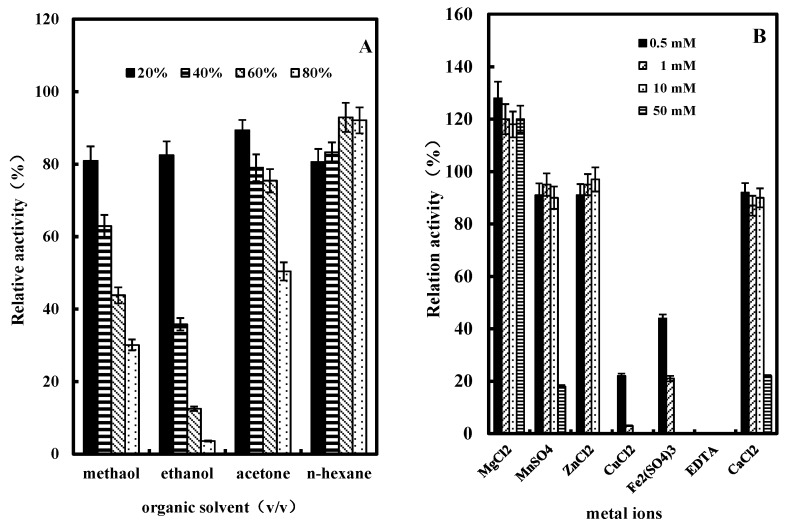
Effects of organic solvents and metal ions on the activity of purified GlcDH from *A. globiformis* C224. (**A**) Effect of organic solvents on the activity of purified GlcDH, organic solvents concentrations: 20%, 40%, 60% and 80% (v/v); (**B**) Effect of metal ions on the activity of purified GlcDH, the enzyme activity in the absence of metal ions was taken as 100% activity. Metal ions or EDTA concentrations: 0.5 mM, 1 mM, 10 mM and 50 mM.

Organic solvents including methanol, ethanol, acetone or *n*-hexane at the concentrations of 20%, 40%, 60% and 80% (v/v) were used to investigate their influence on the purified GlcDH from *A. globiformis* C224. As shown in Figure 7A, all the tested organic solvents showed various degrees of inhibition of GlcDH activity. At 20% (v/v) concentration methanol had a significant inhibitory effect with 80.9% of GlcDH relative activity remaining after treatment. With the increase of concentration to 80% (v/v), ethanol showed the highest inhibition on the GlcDH with 3.6% of relative activity remaining. Compared to other solvents, *n*-hexane had the lowest influence on the GlcDH activity, and even stimulated the GlcDH activity from 83.3% to 92.1% at the concentrations from 40% (v/v) to 80% (v/v). The 3D conformation changes and the blocked catalytic activity center in GlcDH are possible causes for this catalytic activity inhibition in these organic solvents.

#### 2.3.6. Effect of Metal Ions or EDTA on GlcDH Activity

Figure 7B presents the effect of metal ions such as Mg^2+^, Zn^2+^, Mn^2+^, Cu^2+^, Fe^3+^, Ca^2+^ or the ligand EDTA on the activity of purified *A. globiformis* C224 GlcDH. Mg^2+^ had an enhancing effect on the purified GlcDH activity with an increase of about 20%. Zn^2+^, Mn^2+^ or Ca^2+^ at low concentrations of 0.5, 1 and 10 mM had a slight negative effect on the GlcDH activity with inhibition rates of about 3% and 10%, while Cu^2+^ or Fe^3+^ at concentrations of over 10 mM almost completely inhibited the GlcDH activity. EDTA at a concentration of 0.5 mM also completely inhibited the *A. globiformis* GlcDH activity. Therefore, Mg^2+^ is possibly a cofactor on *A. globiformis* GlcDH activity.

## 3. Experimental Section

### 3.1. Chemicals

*N*-Methylphenazonium methyl sulfate (PMS), 2,6-dichlorophenol indophenol (DCIP), polyethylene glycol 6000 (PEG 6000) and D-glucose were purchased from Sinopharm Chemical Reagent CO., Ltd (Shanghai, China). Triton X-100 and Triton X-114 were provided by Sangon Biotech (Shanghai, China). Premixed protein marker was obtained from Takara Bio (Dalian, China). All other chemicals were of analytical grade.

### 3.2. Microorganism and Medium

*A. globiformis* C224 was mutated and screened from *A. globiformis* K1022 by UV mutagenesis [7,27]. The slant stock media contained beef extract 5.0 g/L, peptone 10.0 g/L, NaCl 5.0 g/L and agar 20.0 g/L. Seed media consisted of glucose 20.0 g/L, corn steep liquor 10.0 g/L, urea 2.0 g/L, KH_2_PO_4_ 2.0 g/L and MgSO_4_·7H_2_O 0.5 g/L. Fermentation media for obtaining maximum cell concentration contained glucose 162.0 g/L and corn steep liquor 17.0 g/L. 45.0 g/L of CaCO_3_ was added to the seed and fermentation media for balancing the broth pH.

### 3.3. Preparation of Cell Culture

The slant stock culture was diluted with sterilized water, inoculated into 50 mL of seed medium in a 500 mL Erlenmeyer flask and incubated at a 30 °C for 20 h. This culture at the concentration of 2% (v/v) was inoculated into a 5-L fermenter (GRCB, Green Bio-engineering Co., Ltd, Zhenjiang, China) with working volume of 3 L to prepare the *A. globiformis* cells. Cells were harvested when the OD_650_ value of broth reached to 0.65–0.70 (diluted 20-fold with 1 M HCl). The broth was centrifuged at 10,000× *g*, 4 °C for 20 min to collect *A. globiformis* cells. The collected cells was washed with buffer A (10 mM potassium phosphate buffer containing 5 mM MgCl_2_·6H_2_O, pH 6.0), and then frozen at −20 °C until use.

### 3.4. Extraction and Purification of GlcDH from A. globiformis C224

Frozen cells were suspended in buffer B (10 mM Tris-HCl, containing 1% (w/v) Triton X-114 and 150 mM NaCl, pH 7.4), and extracted with ultrasonic disrupter (Bilon, Shanghai, China) for 60 min. The supernatant containing GlcDH was collected after centrifugation at 16,000× *g*, 4 °C for 30 min, and divided into the hydrophobic and hydrophilic fractions by Triton X-114 phase separation (incubating the supernatant for 10 min at 37 °C). The Triton X-114 phase (hydrophilic fractions) was obtained by centrifuging at 13,000× *g*, 25 °C for 5 min [17].

The obtained Triton X-114 phase was precipitated with five volumes of ice-cold acetone. The precipitate was dissolved in the buffer C (10 mM potassium phosphate buffer containing 5 mM MgCl_2_, 1% (w/v) of Triton X-100 and 1 M of potassium chloride, pH 6.0) [28], mixed with polyethylene glycol 6000 and centrifuged at 10,000× *g* for 20 min to collect the supernatant. The ethanol precipitate fraction was obtained by adding 40% (v/v) of ethanol, dissolved in the buffer D (10 mM sodium phosphate buffer containing 5 mM MgCl_2_ and 1% (w/v) of Triton X-100, pH 7.0), and then applied to a hydroxylapatite column (5 mL, BIO-RAD, Hercules, CA, USA) with the stepwise elution of the buffer D, buffer E (50 mM sodium phosphate buffer containing 5 mM MgCl_2_ and 1% (w/v) of Triton X-100, pH 7.0) and buffer F (100 mM sodium phosphate buffer containing 5 mM MgCl_2_ and 1% (w/v) of Triton X-100, pH 7.0). The elution fractions having GlcDH activity were collected, concentrated by ultrafiltration (Millipore, Hercules, MA, USA), and identified with SDS-PAGE as the purified sample.

### 3.5. Polyacrylamide Gel Electrophoresis

Sodium dodecyl sulfate-polyacrylamide gel electrophoresis (SDS-PAGE) was carried out according to the method of Laemmli [29]. Briefly, purified GlcDH was dissolved in the distilled water and mixing with SDS-PAGE sample loading buffer containing DTT to reduce the protein. Then the GlcDH molecular weight was obtained by comparing and calculating its electrophoretic mobility with those of standard molecular weight markers (Phosphatase b: 97,200 Da, Bovine serum albumin: 66,409 Da, Ovalbumin: 44,287 Da, Carbonic anhydrase: 29,000 Da and Trypsin inhibitor: 20,100 Da).

### 3.6. MALDI-TOF-MS Analysis

The bands of GlcDH in SDS-PAGE were excised, rinsed three times with ultrapure water, and destained with 200 μL 25 mM NH_4_HCO_3_ solution containing 50% CH_3_CN (destaining solution). The gel slices were vacuum-dried, and digested at 37 °C overnight with trypsin. The trypsin hydrolysate containing peptide mixture was combined and freeze-dried for MALDI-TOF-MS analysis with AB 5800 (AB Sciex, Framingham, MA, USA). Spectra mass peaks with the range from 1000 to 2500 m/z were singled out for MS/MS analysis. The peptide mass finger-printings of GlcDH and MS/MS date were obtained and processed using Mass-Lynx V4.1 software (Waters, Milford, MA, USA), and subsequently were converted to PKL files by the Protein-Lynx 2.2.5 software (Waters). The PKL files were analyzed using the MASCOT search engine. The search parameters were confirmed as follows: database, NCBInr; enzyme, Trypsin; taxonomy, Homo sapiens; and allowance of one missed cleavage.

### 3.7. Assay of GlcDH Activity and Protein Determination

The GlcDH activity was measured with ferricyanide as electron acceptor by the method of Wood *et al.* [30]. The reaction mixture contained 10 µmol of potassium ferricyanide, 0.7 mL of Mcllvaine buffer (pH 3.0), 100 µmol of D-glucose and enzyme solution in a total volume of 1.0 mL. The reaction was ended by adding 0.5 mL of the ferric-Dupanol reagent and 3.5 mL of water, and measured OD_660 nm_ value. One unit of GlcDH activity was defined as the amount of GlcDH catalyzing the oxidation of 1 µmol of d-glucose per min, and 4.0 of OD_660 nm_ value was equal to 1 µmol of oxidized d-glucose. Protein content was determined by the Bradford Protein Assay Kit (Sangon Biotech) with bovine serum albumin as standard.

### 3.8. Enzymatic Properties

#### 3.8.1. Substrate Specificity

To determine the substrate specificity of GlcDH, various substrates including d-glucose, d-gluconate, maltose, d-sorbose, d-galactose, d-mannose, d-fructose, d-arabinose, malic acid, sucrose, citric acid or d-xylose (all obtained from Sinopharm Chemical Reagent CO., Ltd.), were separately added to the reaction solution to the concentration of 33 mM.

#### 3.8.2. Effect of pH, Temperature, Organic Solvents or Metal Ions on GlcDH Activity

In order to investigate the effect of pH on the activity of purified GlcDH, eight pHs in the range of 3.0–10.0 were used with the Mcllvaine buffer. The pH stability of GlcDH was determined by incubating the enzyme in the pH range from 3.0 to 10.0 for 48 h at 4 °C and measuring the residual activity.

The optimum catalytic temperature of GlcDH was selected by determining the enzyme activity in the range of 15–85 °C. The temperature stability was determined by measuring the residual activity at various temperatures (20–70 °C) in buffer A for 120 min without adding substrate.

In order to determine the effect of organic solvents on GlcDH activity, methanol, ethanol, acetone or n-hexane was used, organic solvents were added to the reaction solution to the concentration of 20%, 40%, 60% and 80% (v/v), respectively. For assaying the effect of metal ions or metal chelating reagent on GlcDH activity, MgC1_2_, CuCl_2_, ZnCl_2_, Fe_2_(SO_4_)_3_, MnSO_4_ or CaC1_2_ at the concentrations of 0.5, 1, 10 and 50 mM were used. The metal chelating reagent EDTA at the concentrations of 0.5, 1, 10 and 50 mM were added and incubated for 60 min at 4 °C to investigate its influence on GlcDH activity.

### 3.9. Statistical Analysis

Each experiment was repeated three times using duplicate samples. The results were expressed as means ± standard deviations. Statistical comparisons were made by one-way analysis of variance (ANOVA), followed by Duncan’s multiple-comparison test. Differences were considered significant when the *p*-values were <0.05.

## 4. Conclusions

This is the first report on the purification and characterization of a GlcDH from the 2KGlcA industrial producer *A. globiformis* C224. The purified GlcDH showed a molecular weight of 87 kDa and specific activity of 88.1 U/mg. The GlcDH followed a ping-pong mechanism and maintained the highest activity at pH 5.0 and 45 °C. Organic solvents, some metal ions or EDTA can adversely affect GlcDH activity. Our findings to elucidate the structural/enzymic characteristics of *A. globiformis* GlcDH provided us with new insights into improving 2KGlcA industrial stains used in the erythorbic acid industry. Further studies are underway in our laboratory to improve GlcDH properties such as transformation rate, reduce the production period and to find a wider range of production temperatures.
